# The Effect of Combined Decongestive Therapy and Pneumatic Compression Pump on Lymphedema Indicators in Patients with Breast Cancer Related Lymphedema

**Published:** 2012-04-01

**Authors:** M Moattari, B Jaafari, A Talei, S Piroozi, S Tahmasebi, Z Zakeri

**Affiliations:** 1 Department of Nursing, College of Nursing and Midwifery, Shiraz University of Medical Sciences, Shiraz, Iran; 2 Department of General Surgery, Shiraz University of Medical Sciences, Shiraz, Iran; 3 Department of Physiotherapy, College of Rehabilitation, Shiraz University of Medical Sciences, Shiraz, Iran; 4 Cancer Research Center, Shiraz University of Medical Sciences, Shiraz, Iran

**Keywords:** Breast cancer, Lymphedema, Decongestive therapy, Pneumatic compression pump

## Abstract

**Background:**

Lymphedema treatment is difficult and there is no consensus on the best treatment. This study evaluated the effect of combined decongestive therapy (CDT) and pneumatic compression pump on lymphedema indicators in patients with breast cancer related lymphedema (BCRL).

**Methods:**

Twenty one women with BCRL were enrolled. The volume difference of upper limbs, the circumference at 9 areas and shoulder joint range of motion were measured in all patients. CDT was done by an educated nurse in two phases. In first phase, CDT was accompanied by use of a compression pump for 4 weeks, 3 days per week. In second phase, CDT was performed daily without compression pump for 4 weeks by patients at home. At the end of each phase, the same primary measurements were done for patients.

**Results:**

The mean volume difference of the upper limbs and mean difference in circumference in all areas at different phases decreased significantly. Mean flexion, extension, abduction and external rotation (in degrees) at different phases increased significantly.

**Conclusion:**

CDT significantly reduced mean volume and mean circumference of the affected limb, and significantly increased shoulder joint range of motion. The findings support the optimal effects of CDT in the treatment of secondary lymphedema of upper extremity.

**Clinical trial registration number:**

138902212621N8

## Introduction

Patients with breast cancer undergo surgical treatment (mastectomy) accompanied by axillary node dissection and radiotherapy. These treatments impair lymphatic drainage of the affected upper limb, and place patients at risk for secondary lymphedema, which has been reported at rates ranging from 10.0% to 49.0%. Breast cancer-related lymphedema (BCRL) is defined as edema in the upper extremity, due to the insufficient drainage of lymph -a serious and disabling complication of breast cancer treatment. This disorder, which often appears 18-24 months after breast cancer treatment is gradual, chronic and progressive and also resistant to treatment.[1] The incidence of lymphedema after breast cancer treatment, is variable and has been reported between 0.2% and 65.0%.[2][3][4] Also Different diagnostic criteria for lymphedema resulted in different reported prevalence in different studies. On average, of each 4-6 women who undergo breast cancer treatment, one suffers from lymphedema.[1] The US National Cancer Association has estimated the prevalence of lymphedema following metastatic breast cancer to be 9.6 million persons, of which 61.0% (5.86 million) aged over 65 years old.[5] Despite current models for the treatment of breast cancer, lymphedema has remained a major health problem for these patients.[6] Lymphedema treatment is difficult and requires long-term or lifelong physical therapy.[7] For many years, the treatment of this disorder has been controversial,[8] and the literature contains no evidence to suggest the most effective treatment for secondary lymphedema.[9] Many specialists believe that treatment of most types of lymphedema should be primarily conservative.[10] Keeping the upper limb elevated, exercise, compression sleeve, massage therapy, combined decongestive therapy (CDT), pneumatic compression pump, surgery, laser therapy, and drug therapy are among the methods used to treat lymphedema.[1] But so far, surgery and drug therapies have not been successful.[11][12] The CDT method was developed initially in the late 1800s by Winiwarter and then modified by the Vodders in the 1930s.[13] In this method, compression bandaging, manual lymphatic drainage (MLD), exercises to increase lymph circulation and skin care are used.[1][12] Use of a compression pump is not a common part of CDT, but could be used as a complementary treatment.[6]

Several case series have indicated the efficiency of CDT in reducing lymphedema and the circumference of the affected limb; however, sufficient evidence to support this therapeutic method is so far unavailable.[14]

Nurses, as important members of the rehabilitation team, play an important role in the prevention, educa-tion and treatment of patients who survive breast cancer, and can also play an active role in the education, diagnosis and prevention of complications. Thanks to their knowledge of lymphedema, nurses can provide rapid and timely interventions to reduce the severity of lymphedema in these patients.[15][16] The increasing rates of diagnosis and treatment of breast cancer are resulting in an increase in the incidence of lymphedema in these patients. Accordingly, rehabilitation services provided at lymphedema clinics and research regarding treatment for this complication is needed. Previously, patients with lymphedema in our setting had little access to centers in Tehran for treatment. The personal expense and psychological pressure involved in obtaining information and receiving treatment, remain considerable. Moreover, therapeutic specialists lacked the necessary technical and practical expertise to manage lymphedema. Only one study related to treatment of lymphedema has appeared in Iran.[17] Therefore, the aim of this follow up study was to investigate the effect of CDT in combination with compression pump therapy on lymphedema indicators including circumference, volume and range of shoulder joint motions in the upper limb affected by BCRL.

## Materials and Methods

This follow up study involved women referred to the Shahid Mottahari Therapeutic Center in Shiraz be-tween October 2009 and December 2009 (southwestern Iran). Initially 21 patients who met the inclusion criteria agreed to participate and provided their informed consent in writing. The inclusion criteria were a diagnosis of unilateral breast cancer, history of surgery, chemotherapy and (if required) hormone replacement-therapy and radiotherapy, a diagnosis of lymphedema based on assessment by a specialist, mild to severe degree of lymphedema at least 1 year elapsed since axillary node dissection, no being exposed or received CDT, telephone access and age between 35 and 70 years. Before the intervention, we measured the circumference and volume of both upper limbs and joint range of motion in the affected shoulder in all patients. All measurements were done between 8.00 and 14.00 hours.

Upper limb edema was investigated by measuring the circumference of both upper limbs at 9 areas (as previously done by study 10) with a retractable, fiberglass, 150-cm measuring tape and calculating the difference. Measurements were made at the olecranon, 10, 15 and 20 cm above and below the olecranon, at the wrist and at the metacarpophalengeal joint. All measurements were made by the same investigator (a nurse) who used the same procedure at all times. Measurements were recorded as the baseline data, 4 and 8 weeks after treatment.

Upper limb volume was measured by water volume displacement. This method is as the "gold standard" for volumetric measurements and determining volume reduction in patients with lymphedema.[16] Volumes were measured in a pair of specially-constructed cylindrical plexi-glass tanks, each with two drainage taps. The main (internal) tank measured 70.0 cm in height by 21.0 cm in diameter. The external tank measured 60 cm in height and 31.0 cm in diameter. The section area of the internal tank was 330.0 cm². The internal tank was filled with water to a height of 70.0 centimeters. The outer wall of the external tank was marked in centimeters and millimeters to measure the height of the water that over-flowed from the inner tank. The patient stood next to the device and immersed her straightened healthy hand and arm into the inner tank up to a point 15.0 centimeters above the olecranon. The height of the displaced water and that spilled into the outer tank was recorded in centimeters. The patient then removed her healthy upper limb and immerses her affected hand and arm into the internal tank, and the height of the displaced water was again recorded. The difference between the two measurements was multiplied by the section area of the inner tank (330.0 cm) to calculate the volume of water in milliliters displaced by the arm with lymphedema compared to the unaffected limb.

The range of flexion, extension, abduction, adduction and external rotation in the affected shoulder joint was measured with a standard goniometer (based on degree) by the same researcher in all women before CDT therapy and 4 and 8 weeks after. To ensure accuracy flexion, extension, abduction and adduction were measured with the patient in standing position and external rotation of the shoulder joint was measured with the patient in prone position. Patients received treatment with CDT and compression pumping. The accomplishment of the combined decongestive therapy techniques was conducted by one of the researchers and concerning the conduction of these techniques, no expenses was taken from the patients.

The first phase (therapeutic phase) of CDT was done at the clinic where compression pumping was also used to reduce upper limb volume. Each patient received 3 weekly sessions during 4 weeks and each session lasted 60-90 min. In each session, one of the researchers first performed MLD for 30-40 min and then affected upper limb was placed in the compression pump for 15 min. Then the affected limb was bandaged with multilayer compression bandages and remedial exercises were done to increase the lymph circulation. During these sessions, written and verbal information were provided regarding skin and nail care, care of the bandaging and practical training in MLD, how to bandage the upper limb and remedial exercises, and they were prepared for the second phase (maintenance phase), which consisted of long-term self-care to maintain the limb size. To ensure compliance with the instructions and advice regarding self-care, the patients were asked to perform these techniques by themselves during sessions in the second half of the first phase of intervention (third to fourth weeks). During the second treatment phase, patients performed CDT daily at home for a period of 4 weeks. They were given a telephone number to contact for help at any time, and during the second phase, each patient was contacted weekly by telephone by the second author to ask whether she needed any help with her self-care. Each patient completed a daily checklist on selfcare, and use of the checklist was verified during the weekly telephone contact.

At the end of each phase (4 and 8 weeks after starting therapy), displaced water volume, limb circumference and shoulder joint range of motion in the affected upper limb were measured again. The data were analyzed with analytical and descriptive statistics in SPSS software (version 17.0, Chicago, IL, USA). Correlation coefficient between the two methods of volume and circumferential measurements of the upper limbs were examined by Pearson correlation test. Descriptive statistics included absolute and percentage frequencies, means and standard deviations. Mean values of data (displaced water volume, limb circumferences in 9 areas of the affected limb minus the same measures in unaffected limb and the degree of shoulder joint range of motion in the affected upper limb) at different phases of study were compared with a repeated measurement design.

## Results

Findings of the study were presented in four tables. Demographic data of the patients were presented in Table 1. Results of correlations between two methods of measurement were shown in Table 2. Upper limb circumference and limb volume were shown in Table 3. Range of shoulder joint motions was presented in Table 4.

**Table 1:** Characteristics of the patients.

**n=21**	
Age (years)	
Mean±SD	35-70
Range	50.38±9.92
Educational level	
Illiterate	4.8%
Late primary education (from 12 to 14 years)	14.3%
Secondary education	47.6%
University education	33.3%
Marietal status	
Single	4.8%
Married	95.2%
Type of operation (%)	
Breast-preserving procedures	42.9
Modified radical mastectomy	38.1
Radical mastectomy	19
Lymph node removed (number) mean±SD	3.28±3.60
Degree of lymphedema	
Degree 1	0
Degree 2	47.6
Degree3	52.4
Affected arm (%)	
Dominant	52.4
Non-dominant	47.6
Non-Surgical Treatments (%)	
HRT^a^	81
RT^b^	95.2
CT^c^	100

^a^ HRT=Hormone-replacement therapy

^b^ RT=Radiotherapy

^c^ CT=Chemotherapy

**Table 2:** Correlations between the mean difference in circumferences and mean volume displacement of both upper limbs before the intervention.

**Mean difference in circumferences of both upper limbs before the intervention (based on cm)**	**Mean volume difference of both upper limbs before the intervention based on mL (n=21)**	
****	***P *value**	**Pearson correlation **
Wrist	0.004	0.597
Metacarp	0.014	0.528
Below 20	0.001	0.870
Below 15	0.001	0.852
Below 10	0.001	0.800
Olecranon	0.001	0.920
Above 10	0.001	0.808
Above 15	0.001	0.900
Above 20	0.001	0.777

**Table 3:** The comparison of the affected arm edema at different phases of study

**Affected arm edema**	**Before treatment ** **mean±SD**	**4 Weeks ** **mean±SD**	**8 Weeks ** **mean±SD**	***P *value**
Circumferential measurement (cm):				
MCP^a^	1.63±1.56	0.87±1.29	0.72±0.80	0.001
Wrist	2.01±1.69	1.28±1.03	1.25±1.12	0.014
Olecranon	4.56±2.35	3.21±1.81	2.50±1.73	0.001
10↓	5.21±3.06	3.63±2.08	3.45±1.96	0.001
15↓	5.93±3.34	3.95±2.24	3.92±2.44	0.001
20↓	5.24±3.13	3.57±2.21	3.32±2.23	0.001
10↑	4.40±2.90	3.32±2.31	3.29±1.97	0.022
15↑	3.74±2.91	2.69±2.04	2.62±1.72	0.006
20↑	2.83±2.23	1.96±1.60	1.66±1.54	0.001
Volumetric measurement (mlt)	802.23±479.68	561.00±303.48	590.85±362.38	0.001

^a^ MCP: Metacarpophalengeal, statistically significant p< 0.05.

**Table 4:** The comparison of the mean of shoulder joint motions at different phases of study.

**Range of motion (°)**	**Before treatment mean±SD**	**4 Weeks mean±SD**	**8 Weeks mean±SD**	**P value^a^**
Flexion	144.76±11.33	153.38±10.55	151.28±11.53	0.001
Extention	46.04±7.74	51.57±5.37	51.19±5.45	0.004
Abduction	153.23±11.25	161.52±9.76	158.71±8.70	0.001
Adduction	0.47±2.18	0.00±0.00	0.00±0.00	Not done
External rotation	67.71±14.00	78.33±10.87	83.76±8.61	0.001

^a^ Statistically significant p<0.05.

Before intervention, the correlation coefficient between two methods of volume and circumferential measurements of the upper limbs were examined, and it was found that correlation coefficient between two methods of measuring was more than 0.8 in all areas except for 20 cm above the olecranon, wrist and metacarp which was 0.77,0.6 and 0.53 respectively.

The mean difference in circumference between the two upper limbs at different phases of study decreased significantly at all levels including metacarpophalengeal, wrist, 10 cm below olecranon, 15 cm below olecranon, 20 cm below olecranon, olecranon, 10 cm above olecranon, 15 cm above olecranon and 20 cm above olecranon (p<0.05) (Table 3).

The differences in mean volume between the two upper limbs 4 and 8 weeks after the intervention were smaller than before treatment (p<0.001) (Table 3).

Mean range of flexion (p<0.001), extension (p<0.004), abduction (p<0.001) and external rotation (p<0.001) increased 4 and 8 weeks after treatment (Table 4).

Due to the fact that, at 4 and 8 weeks after starting intervention, all amounts related to adduction motion of shoulder joint have become zero therefore, test was not carried out (of course, the normal amount of adduction of shoulder joint is zero degree and could be stated that, the mean of adduction motion of shoulder joint in patients participated in this research before beginning of intervention also has been near to normal range and shoulder joint adduction motion in patients with breast cancer related lymphedema as compare with other shoulder motions suffer lesser from this disorder).

## Discussion

Upper limb lymphedema is a common complication of breast cancer treatments.[18] At present, CDT is a popular, conservative treatment for lymphedema; however, the relative efficacy of its different components has not been investigated.[12] In the present study, the circumference measurements of both upper limbs were carried out at 9 areas, however in one study, it was emphasized that these measurements should be performed at least at 4 areas including metacarpal joints, wrists, 10 cm below olecranon and 15 cm above olecranon, existence of more than 2 cm difference in each of these 4 areas between two upper limbs considered as lymphedema diagnosis.[6]

Furthermore, using water volume displacement is propounded as a golden standard to evaluate lymphedema,[16] but applying this method along with measuring limb circumference has been emphasized in other studies.[19] Therefore, in present study, both methods were used to evaluate lymphedema. However we found that the correlation coefficient between two methods of volume and circumferential measurements in all 9 areas was significant, ranging from 0.528 to 0.92. It is noteworthy to mention that circumferential measures of limbs usually performed at bony landmarks. In one study,[20] this measurement method was done at wrist, metacarp, 10 cm below and 15 cm above the olecranon. Correlations we found between two methods of measurement was relatively strong in just three of this four areas, and relatively weak in the metacarpal area. Therefore, we suggest further studies for determining the best areas of upper limbs for measurement of lymphedema if volumetric measurement is not practical.

Significant decrease in the mean difference in circumference of both upper limbs (at 9 areas), 4 weeks after treatment and the permanency of this reduction 8 weeks after study at 8 areas indicates the positive effects of maintenance phase as well as therapeutic phase of CDT along with compression pumping and effective education of CDT techniques in the form of a self management program to the patients.

Performed interventions to reduce lymphedema have been various. In most of studies, the object has been the comparison of the effect of various parts of CDT with each other. For example, in a study, the effect of two methods of MLD along with compression bandaging and compression bandaging alone were evaluated. Results indicated that both methods were effective in lymphedema volume reduction.[16] In another study, also the effect of compression bandaging with low elasticity alone and compression bandaging in combination with MLD were compared and results indicated that compression bandaging with low elasticity was an effective treatment to reduce mild and moderate lymphedema volume but, adding MLD to the compression bandage was more effective.[21] In another study, remedial exercise along with compression garments were used.[20] At the end of first month, there was significant decrease in circumference in two areas (wrist and 15 cm above olecranon). But, in the present study, in which CDT along with compression pumping were used, 4 weeks after treatment comparing prior to that, the mean difference in circumference of both upper limbs at all measuring areas decreased significantly. Therefore, considering the similarity of demographic characteristics of under studied groups in these two studies, we may say that, performing CDT accompanied by use of compression pumping exhibited more desirable results comparing with remedial exercise along with compression garments. In the second phase of a study, also the effect of MLD was compared with the effect of compression pumping. The results showed that MLD and compression pumping effectively reduced lymphedema volume and no any statistical significant difference was existed between the obtained results of these two treatment methods.[22] In other studies, the effect of CDT was compared with the effect of CDT along with compression pumping.[17][18][23] In spite of the fact that, these intervention were similar but, obtained contradictory results in such a way that, Szuba et al. (2002) and Szolonky et al. (2002) concluded that, CDT along with compression pumping reduced the limb volume more effectively but, Haghighat et al. (2010) concluded that, CDT alone or in combination with compression pumping reduced the limb volume significantly but, CDT alone exhibited better results.

In other studies, the effect of simple lymphatic drainage (which is performed by patients) was compared with MLD (which is performed by expert therapist). The results of these studies showed that MLD reduced the lymphedema volume more significantly than the simple lymphatic drainage.[24][25] In our study, at the time of 8 weeks after treatment, there was no significant difference in the mean difference in circumference of both upper limbs at all measuring areas comparing with 4 weeks after that. This result represents the continuance of obtained results from therapeutic phase throughout maintenance phase. It should be considered that, as a fact, the most edema reduction rate occurred during the first week of CDT performance and at the end of forth week of therapeutic phase, edema volume reduced less and slower.[26] At the second phase of CDT, if no increase occurs in the reduced size of the limb, patients obtained the desirable and considered results.[27] In a study, the therapeutic phase of CDT was performed 5 days per week for a period of 4 weeks and thereafter, patients were undergone maintenance therapy for a period of one year. The difference in circumference of both upper limbs as well as volume difference of both upper limbs was measured at the end of therapeutic phase, 3 months, 6 months and one year after beginning of intervention. Results indicated that, 3 months after starting intervention (beginning of maintenance phase), difference in circumference of both upper limbs had reduced on an average by 1.5 centimeters at the end of therapeutic phase. Thereafter, slightly increased and till one year after starting intervention remained constant about 1 cm lower than the size of the study beginning. Also, volume difference of both upper limbs, reduced by 138 ml at the end of therapeutic phase and thereafter, slightly increased 3 months after study beginning and till 1 year after starting intervention remained constant about 100 ml lower than the amount of starting intervention.[28]

In our study, the mean difference in circumference of both upper limbs, 8 weeks comparing with 4 weeks after treatment, not only has not been increased but, has been reduced insignificantly. This indicates that, by performing self management program at second phase of treatment, patients could maintain their limb volume reduction. The results of the present study indicated that, the mean volume difference of the upper limbs and mean difference in circumference in all areas measured at different phases decreased significantly. The changes of these two variables are indicating the optimal effects of CDT along with compression pumping in lymphedema treatment.

The results related to the mean difference in volume of both upper limbs, 8 weeks comparing with 4 weeks after starting intervention is similar to the obtained results from circumference measurement of both upper limbs. In a study, it was shown that a significant volume decrease which was obtained at the end of first phase, prolonged 9 months after starting treatment and it was specified that, in patients adapted with treatment (86.0% of patients), 90.0% of volume primary reduction in upper and lower limbs has been preserved and patients not adapted to treatment, lost 33.0% of volume reduction of their limbs.[29]

The obtained results from the measuring of shoulder joint range of motion is indicating that, performing CDT along with compression pumping, improved the range of motions of flexion, extension, abduction and external rotation of shoulder joint in these patients. In our study, the most recovery has been exhibited in external rotation of shoulder joint and it could be concluded that, CDT along with compression pumping was so effective in the improvement of external rotation of shoulder joint that affected with lymphedema. In this regard, it should be taken into consideration that the created limitation in the range of external rotation of shoulder joint comparing with its other motions would be relieved immediately following by CDT.[30] In a study, the effects of CDT and standard physiotherapy in the treatment of lymphedema secondary to breast cancer were compared. After treatment, the range of shoulder flexion and abduction increased significantly in both groups (p<0.05). Results indicated that, although patients of both groups experienced the range of motion increment and lymphedema decrement but, total recovery in the CDT group was more than that of standard physiotherapy group.[12] Although the duration of CDT in their study was similar to our study but, performing CDT in their study resulted in improvement in the shoulder flexion and abduction only. But, the results of present study are indicating to a significant increase in the range of shoulder flexion, extension, abduction and external rotation in such a way that, the range of these motions has reached to the normal amount. Of course, it is suggested to consider the evaluation of shoulder joint range of motion on the basis of comparing the range of motions of joints from both upper limbs with each other in other studies.

CDT along with compression pumping reduced the difference in upper limbs circumference and the difference in volume between the affected and unaffected limb. Patient education in CDT skills can enable them to maintain the reductions in limb measurements achieved by ambulatory treatment. The use of compression pumping together with CDT was effective in increasing the range of shoulder joint motions. Nurses can play a potentially important role in providing lymphedema therapy and patient education and support which can increase the effectiveness of treatment.
